# Elevated Prevalence of Oral HPV Infection Among Females with Periodontitis: A Cross-Sectional Study

**DOI:** 10.3290/j.ohpd.c_2446

**Published:** 2026-01-15

**Authors:** Defeng Liang, Yixun Wang, Yanfen Li, Zhiying Chen, Qing Zeng, Shanshan Ha, Xincai Zhou, Donglei Wu

**Affiliations:** a Defeng Liang Dentist, Department of Stomatology, Shenzhen Baoan Women’s and Children’s Hospital, Jinan University, Shenzhen, China. Study design and concept, conducted the study, wrote and reviewed the manuscript.; b Yixun Wang Postgraduate, School of Public Health, Southern Medical University, Guangzhou, Guangdong, China. Study design and concept, conducted the study, wrote and reviewed the manuscript.; c Yanfen Li Dentist, Department of Stomatology, Shenzhen Baoan Women’s and Children’s Hospital, Jinan University, Shenzhen, China. Study design and concept, conducted the study, wrote and reviewed the manuscript.; d Zhiying Chen Dentist, Department of Stomatology, Shenzhen Baoan Women’s and Children’s Hospital, Jinan University, Shenzhen, China. Study design and concept, conducted the study, wrote and reviewed the manuscript.; e Qing Zeng Dentist, Department of Stomatology, Shenzhen Baoan Women’s and Children’s Hospital, Jinan University, Shenzhen, China. Study design and concept, conducted the study, wrote and reviewed the manuscript.; f Shanshan Ha Dentist, Department of Stomatology, Shenzhen Baoan Women’s and Children’s Hospital, Jinan University, Shenzhen, China. Study design and concept, conducted the study, wrote and reviewed the manuscript.; g Xincai Zhou Dentist, Department of Stomatology, Shenzhen Baoan Women’s and Children’s Hospital, Jinan University, Shenzhen, China. Study design and concept, conducted the study, wrote and reviewed the manuscript.; h Donglei Wu Dentist, Department of Stomatology, Shenzhen People’s Hospital, Shenzhen, China. Study design and concept, conducted the study, wrote and reviewed the manuscript.

**Keywords:** cross-section study, female, oral HPV infection, periodontitis

## Abstract

**Purpose:**

This study investigated the association between periodontitis and oral HPV infection, while exploring the role of oral bacterial microbiota diversity.

**Methods and Materials:**

Data from 4,685 adults in the NHANES 2009–2012 cycles were analysed. Periodontitis was defined based on clinical examination, and oral HPV infection was identified using PCR from oral rinse samples. Multivariable logistic regression models were employed to assess the relationship, adjusting for body mass index (BMI), age, sex, ethnicity, education, smoking, alcohol consumption, daily dental flossing, and history of systemic diseases. Subgroup analyses were stratified by age, sex, and education. Mediation analysis was performed to evaluate whether the oral microbiome acts as a mediator in the relationship between periodontitis and oral HPV infection.

**Results:**

No statistically significant overall association was found between periodontitis and oral HPV infection (P > 0.05). However, females with moderate to severe periodontitis exhibited increased odds of oral HPV infection (P < 0.05). Oral HPV infection was associated with greater microbial diversity (higher operational taxonomic units [OTUs]). No significant mediating effect of the oral microbiome was observed.

**Conclusion:**

Moderate to severe periodontitis appears to be associated with higher odds of oral HPV infection in females. These findings highlight the potential relationship between oral health, microbial diversity, and oral HPV infection.

**Clinical Implication:**

In the general population, periodontitis does not appear to be a major risk factor for oral HPV; however, female with moderate to severe periodontitis and individuals with higher educati-on showed increased odds of oral HPV infection, suggesting that maintaining periodontal health may be particularly important for HPV related risk management in these subgroups.

Oral human papillomavirus (HPV) infection is a major factor contributing to the rising incidence of oropharyngeal and oral squamous cell carcinomas (OPSCC and OSCC, respectively).^19,21,26^ Approximately 70% of oropharyngeal cancers are attributed to HPV infection , with an oral HPV prevalence of 7.7% among adults.^15,35,43^ While many infections resolve spontaneously, persistent HPV significantly increases cancer risk.^10,11^ However, factors influencing viral persistence remain incompletely understood.

The human microbiome appears to play a significant role in HPV infection dynamics. Studies have demonstrated that dysbiosis in vaginal and cervical microbiota increases HPV susceptibility and delays clearance.^5^ Similarly, gut microbial imbalances have been linked to compromised immunity and increased HPV persistence risk.^44^ In addition, oral microbial dysbiosis is associated with viral infection.^14^ These findings suggest that microbial communities across body sites, including the oral cavity, may influence local immune environments and HPV outcomes.

Chronic inflammation also affects HPV infection.^28^ Elevated inflammatory markers like TNF-α correlate with reduced HPV clearance.^18^ Systemic inflammatory conditions—including diabetes, cardiovascular disease, and autoimmune disorders—are associated with altered immune responses that may increase HPV vulnerability.^13,16,22^ Notably, the persistence and clearance of oral HPV infection are closely related to oral inflammatory status.^3, 18^ Periodontitis, characterised by chronic oral inflammation and bacterial dysbiosis, potentially contributes to both local and systemic inflammatory burden. Recent research suggests a link between periodontitis and oral HPV infection, even in vaccinated individuals.^23^ Periodontal pockets may serve as HPV reservoirs, while dysbiotic oral microbiota could compromise mucosal barriers and antiviral responses.^4^ Despite these observations, the relationships among periodontitis, the oral microbiome, and oral HPV infection remain poorly characterised, particularly regarding how oral microbial diversity might influence HPV infection likelihood. In this crosssectional study, we hypothesised that periodontitis – a state of chronic gingival inflammation and oral microbial dysbiosis – is associated with a higher prevalence of oral HPV infection. We further explored whether oral microbiome diversity could help explain this association. Clarifying these associations may underscore the importance of periodontal health in HPV‑related risk management.

## METHODS AND MATERIALS

### Data Extraction and Study Population

The National Health and Nutrition Examination Survey (NHANES, https://www.cdc.gov/nchs/nhanes/index.html) is a cross-sectional, national research programme designed to assess the health and nutritional status of individuals in the United States. Data from the NHANES 2009–2012 cycles were used to assess the association between the presence of periodontitis and oral HPV infection. The NHANES employs a complex, stratified multistage probability sampling methodology to assess the non-institutionalised US population. Each biennial survey cycle systematically collects comprehensive health-related data. For this research, we aggregated and analysed demographic, questionnaire, periodontal examination and laboratory examination data from two consecutive NHANES cohorts spanning 2009–2010 and 2011–2012. The procedures and methodologies employed by NHANES are accessible on its official website. All participants provided informed consent, and the data collection procedures were approved by the National Center for Health Statistics (NCHS) Research Ethics Review Board. Further details, including protocol numbers, are documented at: https://www.cdc.gov/nchs/nhanes/irba98.htm.

### Inclusion Criteria

Individuals aged 30 years and older were eligible for a full-mouth periodontal examination if they had at least one natural tooth and did not have any medical condition necessitating antibiotic prophylaxis prior to periodontal probing. In addition, individuals who reported having undergone a heart transplant, having an artificial heart valve, or congenital heart disease (excluding mitral valve prolapse) were excluded from participating in the periodontal examination (https://wwwn.cdc.gov/Nchs/Data/Nhanes/Public/2011/DataFiles/OHXPER_G.htm).

### Definition of Periodontitis

The assessment of periodontal status was conducted through a comprehensive full-mouth periodontal examination (FMPE) based on the NHANES protocol, evaluating six sites per tooth across all non-third molar teeth in the mobile examination centre (MEC). Measurements of gingival recession and probing pocket depth were obtained at six sites per tooth using a colour-coded Hu-Friedy periodontal probe. Periodontitis was defined according to the classification criteria set by the Centers for Disease Control and the American Academy of Periodontology (CDC/AAP).^12^ The mild periodontitis was defined as either one site with a probing depth (PD) of 5 mm or more, or at least two interproximal sites with 3 mm or more in attachment loss (AL), and at least two interproximal sites with a PD of at least 4 mm, not occurring on the same tooth. The definition for moderate periodontitis included at least two interproximal sites with PD of at least 5 mm, not on the same tooth, or at least two interproximal sites not on the same tooth with an AL of at least 4 mm. Severe periodontitis was characterised by a PD of at least 5 mm at one interproximal site, along with at least two interproximal sites on different teeth showing AL of at least 4 mm (Supplementary Table 1). The study data were dichotomised into categories of mild, moderate, or severe periodontitis versus no periodontitis, considering the available sample size.

**Table 1:** Characteristics of the participants at baseline

Variables	Total (n = 4685)	HPV negative (n = 4302)	HPV positive (n = 383)	P
**Age**				0.14
median (IQR)	48.00 (39.00, 57.00)	47.00 (39.00, 56.00)	50.00 (40.00, 57.00)	
**Sex, n (%)**			< 0.0001
female	2265 (48.47)	2187 (50.74)	78 (20.35)	
male	2420 (51.53)	2115 (49.26)	305 (79.65)	
**Ethnicity, n (%)**				0.17
black	1091 (11.52)	974 (11.22)	117 (15.26)	
Mexican	788 (8.72)	722 (8.70)	66 (9.01)	
white	1804 (67.39)	1668 (67.59)	136 (64.83)	
other	1002 (12.37)	938 (12.48)	64 (10.90)	
**Education, n (%)**				0.08
< 9th grade	468 (5.06)	430 (5.08)	38 (4.87)	
9th–12th grade	1664 (30.91)	1502 (30.35)	162 (37.86)	
> ty12th grade	2553 (64.03)	2370 (64.57)	183 (57.27)	
**Body mass index (BMI)**			0.75
median (IQR)	28.10 (24.71, 32.50)	28.05 (24.70, 32.50)	28.20 (25.30, 32.65)	
**Smoke, n (%)**			< 0.0001
no	2601 (55.28)	2462 (56.87)	139 (35.46)	
yes	2084 (44.72)	1840 (43.13)	244 (64.54)	
**Alcohol consumption, n (%)**		0.002
no	549 (8.84)	524 (9.22)	25 (4.10)	
yes	4136 (91.16)	3778 (90.78)	358 (95.90)	
**Diabetes mellitus (DM), n (%)**			0.34
no	3488 (79.16)	3219 (79.49)	269 (75.15)	
Prediabetes	370 (8.24)	333 (8.01)	37 (11.03)	
DM	827 (12.60)	750 (12.50)	77 (13.82)	
**Cardiovascular diseases (CVD), n (%)**			0.03
no	4381 (94.42)	4033 (94.74)	348 (90.53)	
yes	304 (5.58)	269 (5.26)	35 (9.47)	
**Hypertension, n (%)**			0.61
no	2827 (64.00)	2605 (64.17)	222 (61.96)	
yes	1858 (36.00)	1697 (35.83)	161 (38.04)	
**Floss daily, n (%)**				0.41
no	3241 (69.62)	2963 (69.38)	278 (72.62)	
yes	1444 (30.38)	1339 (30.62)	105 (27.38)	
**Periodontitis, n (%)**		< 0.001
no	2226 (56.63)	2093 (57.97)	133 (39.96)	
mild	318 (6.43)	296 (6.56)	22 (4.85)	
moderate	1536 (28.01)	1378 (27.20)	158 (38.05)	
severe	605 (8.94)	535 (8.27)	70 (17.15)	
**Alpha diversity**
**Operation taxonomic units**		0.001
median (IQR)	122 (98.00, 151.00)	121 (97.00, 151.00)	131 (110.0, 159.00)	
**Faith’s phylogenetic diversity index**			0.01
median (IQR)	13.93 (12.10, 16.61)	13.89 (12.03, 16.58)	14.44 (12.80, 16.73)	
**Shannon-Wiener index**			0.04
median (IQR)	4.63 (4.23, 5.03)	4.63 (4.23, 5.02)	4.74 (4.24, 5.12)	
**Inverse Simpson index**			0.06
median (IQR)	0.92 (0.89, 0.94)	0.92 (0.89, 0.94)	0.92 (0.89, 0.94)	
IQR, interquartile range

### Detection of Oral HPV

Oral rinse specimens from NHANES individuals were collected and sent to the Gillison Laboratory at the Ohio State University, Columbus, Ohio. Following the NHANES Laboratory/Medical Technologists Procedures Manual (LPM), samples were centrifuged, and the pellet was resuspended in 1 ml of Puregene cell lysis solution. Incubation, digestion with DNase-free RNase A, and overnight Proteinase K digestion were performed, followed by heat inactivation. Protein precipitation solution was added, and further purification was conducted using the Puregene DNA purification kit. After DNA purification, a total of 37 HPV types were analysed through a multiplex polymerase chain reaction (PCR) assay targeting the conserved L1 region, which including HPV-6, -11, -16, -18, -26, -31, -33, -35, -39, -40, -42, -45, -51, -52, -53, -54, -55, -56, -57, -58, -59, -61, -62, -64, -66, -67, -68, -69, -70, -71, -72, -73, -81, -82 (MM4 and IS39 subtypes), -83, -84 and -89, and ß-globin (https://wwwn.cdc.gov/Nchs/Nhanes/2011-2012/ORHPV_G.htm).

### Covariables

Demographic and lifestyle characteristics, along with the history of systemic diseases, were gathered through questionnaires administered by trained interviewers using the Computer-Assisted Personal Interviewing (CAPI) system. The demographic questionnaires can be accessed at the following link: NHANES Demographic Questionnaires (ttps://wwwn.cdc.gov/nchs/nhanes/search/datapage.aspx?Component=Examination&CycleBeginYear=2011), including age, sex (female, male), ethnicity (black, Mexican, white and other), education (< 9th grade, 9th–12th grade, > 12th grade), body mass index (BMI), smoking status (yes, no), alcohol consumption (yes, no) and using dental floss daily (yes, no). Hypertension was defined as systolic blood pressure > 140 mmHg or diastolic blood pressure > 90 mmHg. Diabetes Mellitus (DM) diagnosis included clinical assessment, HbA1c > 6.5%, fasting plasma glucose ≥ 7.0 mmol/L, random/two-hour oral glucose tolerance test (OGTT) plasma glucose ≥ 11.1 mmol/L, or documented use of antidiabetic medication/insulin therapy.^40^ Cardiovascular disease (CVD) was identified through self-reported diagnoses, confirmed by medical professionals, encompassing conditions like congestive heart failure, coronary heart disease, angina, heart attack, or stroke.^20^

### Detection of Oral Microbiome

The oral microbiome dataset obtained from the NHANES database is derived from oral rinse samples collected during consecutive survey cycles in 2009–2012. The data set includes alpha diversity metrics and relative abundance information. This study utilised oral rinse samples originally collected to investigate the prevalence of oral human papillomavirus in the US population. The microbiome testing involved PCR amplification of the V4 region of the 16S ribosomal RNA gene from extracted DNA. Sequencing data were processed using QIIME and DADA2 software, with taxonomic classification based on the SILVA version 123 database. This approach provided a comprehensive characterisation of the oral bacterial microbiome. Methodological details for molecular analysis, including DNA isolation, sequencing workflows, and computational processing, are publicly available on the NHANES platform.^39^ Several oral microbiome diversity indices were calculated and used in the analyses to assess microbial diversity and richness. The observed operational taxonomic units (OTUs) represent the species richness in a sample. Faith’s phylogenetic diversity index measures the total branch length spanned by the phylogenetic tree, reflecting the evolutionary diversity of the microbial community. The Shannon–Wiener index accounts for both richness and evenness of species in the community, while the inverse Simpson index is more sensitive to the abundance of dominant taxa. Supplementary Table 2 summarises the key features and references for each index used in this study.

**Table 2:** Univariate regression analysis for the association between periodontitis and oral HPV infection

Character	Estimate	Std. Error	t value	P value	OR	95% CI
**Age**	0.01	0.01	1.35	0.19	1.01	1.01 (1.00, 1.02)
**Sex**						
Female	ref	ref	ref	ref	ref	ref
Male	1.39	0.16	8.68	<0.0001	4.03	4.03 (2.91, 5.59)
**Ethnicity**						
black	ref	ref	ref	ref	ref	ref
Mexican	–0.27	0.21	–1.32	0.20	0.76	0.76 (0.50, 1.16)
white	–0.35	0.18	–1.9	0.07	0.71	0.71 (0.48, 1.03)
ther	–0.44	0.23	–1.91	0.07	0.64	0.64 (0.40, 1.03)
**Education**						
< 9th grade	ref	ref	ref	ref	ref	ref
> 12th grade	–0.08	0.29	–0.27	0.79	0.93	0.93 (0.51, 1.67)
9th–12th grade	0.26	0.29	0.91	0.37	1.3	1.30 (0.72, 2.35)
**BMI**	0	0.01	–0.08	0.94	1	1.00 (0.98, 1.02)
**Smoking**						
no	ref	ref	ref	ref	ref	ref
yes	0.88	0.13	6.55	< 0.0001	2.4	2.40 (1.83, 3.15)
**Alcohol consumption**					
no	ref	ref	ref	ref	ref	ref
yes	0.87	0.26	3.31	0.002	2.38	2.38 (1.40, 4.05)
**Diabetes mellitus**						
no	ref	ref	ref	ref	ref	ref
Prediabetes	0.38	0.31	1.23	0.23	1.46	1.46 (0.78, 2.71)
DM	0.16	0.2	0.77	0.45	1.17	1.17 (0.77, 1.77)
**Periodontitis**						
no	ref	ref	ref	ref	ref	ref
mild	0.07	0.3	0.23	0.82	1.07	1.07 (0.58, 1.98)
moderate	0.71	0.21	3.4	0.002	2.03	2.03 (1.33, 3.10)
severe	1.1	0.32	3.42	0.002	3.01	3.01 (1.56, 5.80)
**Cardiovascular disease**						
no	ref	ref	ref	ref	ref	ref
yes	0.63	0.28	2.3	0.03	1.88	1.88 (1.07, 3.30)
**Hypertension**						
no	ref	ref	ref	ref	ref	ref
yes	0.09	0.18	0.52	0.61	1.1	1.10 (0.76, 1.60)
**Floss daily**						
no	ref	ref	ref	ref	ref	ref
yes	–0.16	0.19	–0.83	0.41	0.85	0.85 (0.58, 1.26)
**Alpha diversity**						
**Operation taxonomic units**	0	0	3.14	0.004	1	1.00 (1.00, 1.01)
**Faith’s phylogenetic diversity index**	0.04	0.01	2.79	0.01	1.04	1.04 (1.01, 1.07)
**Shannon-Wiener index**	0.18	0.08	2.17	0.04	1.2	1.20 (1.01, 1.42)
**Inverse Simpson index**	2.04	1.7	1.2	0.24	7.68	7.68 (0.24, 245.87)
ref, reference.

### Statistical Analysis

For continuously distributed data with normality, means and standard deviation (SD) were reported, while non-normally distributed continuous variables were presented as median and interquartile range (IQR), including age, BMI, OTU, Faith’s phylogenetic diversity index, Shannon–Wiener index, and inverse Simpson index. Categorical variables were expressed as counts (percentages), including sex, ethnicity, education, smoking and alcohol consumption, presence of DM, CVD, periodontitis, and using dental floss daily. The Kruskal-Wallis test was utilised for comparing non-normally distributed continuous variables (eg, age, BMI, OTU, Faith’s phylogenetic diversity index, Shannon–Wiener index, and inverse Simpson index) between individuals with and without oral HPV infection. The Chi-squared test was employed to compare categorical variables (eg, sex, ethnicity, education, smoking and alcohol consumption, presence of DM, CVD and periodontitis, and daily use of dental floss) between those with oral HPV infection and those without. Univariate and multivariate logistic regression analysis were employed to evaluate the independent correlation between the occurrence of periodontitis and oral HPV infection with varying degrees of severity using odds ratios (ORs) and 95% confidence intervals (CIs). Model 1 was adjusted for body mass index (BMI), age, sex, ethnicity, and education level. Model 2 included additional adjustments for smoking status, alcohol consumption, and daily dental flossing, alongside the variables in Model 1. Model 3 further incorporated adjustments for hypertension, cardiovascular disease, diabetes mellitus, OTUs, Faith’s phylogenetic diversity index, Shannon–Wiener index, and Inverse Simpson index, in addition to all variables accounted for in Model 2. A test for trend was employed to assess the dose–response relationship between the severity of periodontitis and the presence of oral HPV infection. Subgroup analyses were conducted stratified by sex, age, and education level using multivariate regression analysis. Additionally, multiplicative interactions among these variables were assessed through likelihood ratio tests. The P value less than 0.05 was considered significant. To explore whether the oral microbiome mediates the association between periodontitis and oral HPV infection, a mediation analysis was conducted using the mediation package in R. The Bootstrap method was applied to estimate the standard error of the mediation effect, ensuring greater robustness in the analysis. All statistical analyses were performed using a frequentist framework. Thus, estimates of association (eg, odds ratios) are presented with 95% CIs.

## RESULTS

### Basic Characteristics of Study Population from NHANES 2009–2012

In this study, a total of 4,685 participants who met the predefined inclusion criteria were analysed. Among these, 4,302 participants were not infected with oral HPV infection, while 383 participants were identified as having oral HPV infection. The study population comprised 2,420 males (51.53%) and 2,265 females (48.47%), with a mean age of 48 years (interquartile range: 39–57). The presence of oral HPV infection was significantly associated with male sex, smoking, alcohol consumption, and a history of CVD or periodontitis (P < 0.05). In addition, a significant difference in alpha diversity was observed among the groups (P < 0.05). Subsequent analyses revealed that individuals with oral HPV infection exhibited a higher number of OTUs and increased overall diversity, as indicated by elevated Shannon–Wiener and Faith’s phylogenetic diversity indices. However, no significant difference was observed in the inverse Simpson index, suggesting comparable evenness in species abundance between the two groups (Table 1).

### Univariate Regression Analysis for the Association Between Periodontitis and Oral HPV Infection

The results of univariate logistic regression analysis indicated significant associations in individuals with the oral HPV infection, indicating positive correlations with male, smoking and alcohol consumption, moderate to severe periodontitis, and CVD, all showing statistical significance (P < 0.05). In addition, the alpha diversity aspects, including OTU, Faith’s phylogenetic diversity index and Shannon–Wiener index, were positively associated with the oral HPV infection (P < 0.05). However, the analysis did not detect significant associations between periodontitis and ethnicity, education, BMI, DM, hypertension or daily dental flossing (P > 0.05) (Table 2).

### Multivariate Regression Analysis for the Association Between Periodontitis and Oral HPV Infection

Table 3 presents the logistic regression analysis on the relationship between periodontitis and oral HPV infection. In the crude model, oral HPV infection showed a positive association with moderate to severe periodontitis (odds ratio [OR]: 2.03, 95% confidence interval [CI]: 1.33, 3.10, P < 0.05 and OR: 3.01, 95% CI: 1.56, 5.80, respectively). However, no significant correlations were observed between periodontitis and oral HPV infection in adjusted Model 1, Model 2 and Model 3 (fully adjusted model) (P > 0.05). Additionally, a statistically significant trend in the association between periodontitis severity and oral HPV infection was observed in the crude model (P for trend < 0.05). Specifically, as the severity of periodontitis increased from moderate to severe, there was a corresponding increase in the odds of oral HPV infection. This trend suggests a potential dose–response relationship, indicating that higher levels of periodontitis may be associated with an increased odds of oral HPV infection. However, this dose–response relationship was not observed in the adjusted models (P for trend > 0.05) (Table 3).

**Table 3:** Multiple logistic regression analysis for the association between periodontitis and oral HPV infection

Periodontitis	Oral HPV infection
Crude model	Model 1	Model 2	Model 3
character	OR (95%CI)	OR (95%CI)	OR (95%CI)	OR (95%CI)
no	ref	ref	ref	ref
mild	1.07 (0.58, 1.98)	0.87 (0.46, 1.65)	0.85 (0.46, 1.59)	0.84 (0.43, 1.66)
moderate	2.03 (1.33, 3.10)*	1.65 (0.99, 2.75)	1.45 (0.86, 2.47)	1.44 (0.80, 2.50)
severe	3.01 (1.56, 5.80)*	1.97 (0.90, 4.34)	1.62 (0.72, 3.63)	1.57 (0.65, 3.82)
P for trend	< 0.001	0.05	0.15	0.21
ref, reference; OR, odds ratio; CI, credibility interval; Model 1 was adjusted for body mass index (BMI), age, sex, ethnicity and education. Model 2 was adjusted for BMI, age, sex, ethnicity, education, smoking, alcohol consumption and daily dental flossily. Model 3 was adjusted for BMI, age, sex, ethnicity, education, smoking, alcohol consumption, daily dental flossing, hypertension, cardiovascular disease, diabetes mellitus, operation taxonomic units, Faith’s phylogenetic diversity index, Shannon–Wiener index and Inverse Simpson index. *P value < 0.05.

### Subgroup Analysis for the Association Between Periodontitis and Oral HPV Infection in a Fully Adjusted Model

Subgroup analyses using fully adjusted Model 3 were conducted to explore the relationship between periodontitis and oral HPV infection across different demographic characteristics. Among the sexes, a significant correlation was observed between moderate to severe periodontitis and oral HPV infection among females, with an odds ratio (OR) of 2.59 (95% CI: 1.04–6.49) for moderate periodontitis and 5.73 (95% CI: 2.02–16.22) for severe periodontitis. Additionally, increasing severity of periodontitis was associated with a marked rise in odds ratios for oral HPV infection, as shown in Table 4 (P for trend < 0.0004). Regarding education, individuals with at least 9 years of education who had severe periodontitis demonstrated a higher odds ratio for oral HPV infection, with ORs of 1.95 (95% CI: 1.12–3.38) for the 9th–12th grade group and 3.22 (95% CI: 1.26–8.25) for those with more than 12 years of education. In the population with 9th–12th grade education, a higher severity of periodontitis was linked to a significant increase in odds ratios for oral HPV infection, as indicated in Table 4 (P for trend < 0.0004). Among age groups, no statistically significant differences in the association were observed among individuals under the age of 50. Interestingly, an interaction between sex and periodontitis (P < 0.05) suggested that sex may influence the effect of periodontitis on oral HPV infection (Table 4).

**Table 4:** Subgroup analysis for the association between periodontitis and oral HPV infection in model 3

Character	No	Mild periodontitis	Moderate periodontitis	Severe periodontitis	P for trend	P for interaction
**Sex**						0.04
Male	ref	0.64 (0.33, 1.24)	1.23 (0.75, 2.03)	1.27 (0.59, 2.74)	0.39	
Female	ref	2.21 (0.65, 7.53)	2.59 (1.04, 6.49) ^*^	5.73 (2.02, 16.22) ^*^	0.004	
**Education**						0.13
< 9th grade	ref	3.15 (0.60, 16.44)	3.54 (0.99, 12.59)	2.17 (0.56, 8.40)	0.09	
9th–12th grade	ref	1.43 (0.52, 3.93)	2.11 (1.14, 3.92) ^*^	1.95 (1.12, 3.38) ^*^	0.004	
> 12th grade	ref	0.75 (0.31, 1.82	1.44 (0.87, 2.37)	3.22 (1.26, 8.25) ^*^	0.04	
**Age**						0.76
=< 50	ref	1.11 (0.54, 2.30)	1.65 (1.00, 2.72)	1.70 (0.67, 4.28)	0.05	
> 50	ref	0.78 (0.20, 2.96)	1.83 (0.81, 4.12)	2.82 (0.82, 9.69)	0.1	
ref, reference; *P value < 0.05

### The Causal Mediation Analysis of Oral Microbiome on the Relationship Between Periodontitis and Oral HPV Infection in Females

The analysis revealed that periodontitis might have a direct impact on oral HPV infection in females (P < 0.05) when examining the total effect across the four oral microbiome indices: OTUs, Shannon–Wiener index, Inverse Simpson index, and Faith’s phylogenetic diversity index. In contrast, the lack of significant differences in the average causal mediation effect (ACME) across all indices of the oral microbiome (P > 0.05) indicates that the oral microbiome does not exert a significant indirect effect on the association between periodontitis and oral HPV infection in females (Table 5).

**Table 5:** The causal mediation analysis of oral microbiome on the relationship between periodontitis and oral HPV infection in female

Oral microbes	ACME	ADE	Total effect	Proportion of mediated
OTU	0.003 (–0.002, 0.010)	0.018* (0.03, 0.030)	0.020** (0.006, 0.040)	0.130 (–0.121, 0.59)
Shannon–Wiener index	0.000 (0.004, 0.000)	0.020* (0.005, 0.040)	0.020** (0.005, 0.040)	0.003 (–0.178, 0.24)
Faith’s phylogenetic diversity index	0.003 (–0.001, 0.010)	0.017* (0.001, 0.030)	0.020** (0.003, 0.040)	0.131 (–0.078, 0.700)
Inverse Simpson index	0.001 (–0.001, 0.000)	0.020* (0.003, 0.040)	0.020* (0.004, 0.04)	0.032 (–0.074, 0.340)
* P value < 0.05; **, P value < 0.01; ACME, average causal mediation effect; ADE, average direct effect; OTU, operational taxonomic units.

## DISCUSSION

In this study, we found no significant association between moderate to severe periodontitis and oral HPV infection after adjusting for all covariates. However, subgroup analyses revealed that females with severe periodontitis had a 5.73-fold higher likelihood of oral HPV infection compared to those with healthy periodontium. Additionally, among individuals with at least 9 years of education, moderate to severe periodontitis was also associated with increased odds of oral HPV infection. Overall, periodontitis was not clearly linked to oral HPV, so routine HPV counselling need not change based on periodontal status alone. However, higher odds were observed in certain subgroups – females with moderate to severe periodontitis and those with higher education – where reinforcing periodontal prevention and treatment alongside standard HPV risk counselling may be prudent. Oral HPV was associated with greater oral microbiome diversity, but diversity did not explain the periodontitis–HPV link, so it has no current clinical role. Prospective studies should confirm these subgroup signals and clarify mechanisms to guide targeted prevention.

Existing literature presents mixed evidence on this topic. A recent meta-analysis indicated a weak but positive association between periodontitis and oral HPV infection,^1^ while other reports remain inconclusive. Consistent with a prior NHANES analysis,^42^ reporting no overall association between periodontitis and oral HPV detected in oral rinses, our primary analysis also found no significant association in the full sample. Importantly, our study adds three advances: (i) severity-based classification of periodontitis, (ii) identification of sex-specific effect modification with a female subgroup signal for moderate to severe disease, and (iii) mechanistic assessment of oral microbiome diversity, which was associated with HPV but did not mediate the periodontitis–HPV association. These analytical dimensions, implemented within NHANES, provide incremental public health insight by pinpointing a potentially higher-risk subgroup – women with moderate to severe periodontitis – and by clarifying that diversity alone is unlikely to explain the observed association. However, other studies have demonstrated an association between oral inflammation and oral HPV infection.^6^ For example, a cross-sectional study of 740 participants found that severe periodontitis was positively correlated with oral HPV infection, with individuals exhibiting clinical attachment loss ≥ 7 mm and pocket depth ≥ 6 mm showing 2–3 times higher odds of HPV infection.^25^ Higher bleeding on probing and increased sites with both PD ≥ 4 mm and bleeding were also observed among HPV-positive adults.^6^ These discrepancies in findings may be explained by differences in sample size, population characteristics, study design, and methodology for assessing oral HPV and periodontitis.

In this study, numerous confounding variables identified from published articles were collected and adjusted for in a multiple logistic regression model. High alcohol consumption is associated with an elevated risk of prevalent genital HPV infections among US men. Our study also confirmed a positive association between alcohol intake and oral HPV infection; however, the biological role of alcohol in oral HPV infection still requires further investigation. A significant disparity in oral HPV infection rates between genders was detected, with male participants showing nearly four times the infection rate of females. This disparity may be attributed to a lower frequency of males being vaccinated before initiating oral sex than females.^2^ Additionally, urban populations exhibited a statistically higher frequency of HPV compared to rural areas in South-Eastern Poland.^17^ Interestingly, in this study, we found that females with moderate to severe periodontitis had a higher likelihood of experiencing oral HPV infection events than males. Moreover, individuals under 50 with oral HPV infection showed a higher likelihood of experiencing periodontitis compared to those aged 50. No studies have explored and confirmed the close relationship between oral HPV infection in women and the presence of an HPV-positive cervix, emphasising the connection to oral sex.^4^ Another study suggests that the highest predicted likelihood of high-risk oral HPV infection occurred in men, black participants, individuals with a daily cigarette consumption exceeding 20 cigarettes, current marijuana users, and those reporting 16 or more lifetime vaginal or oral sex partners.^27^ Smoking has been shown to upregulate the quantity of HPV genome copies and facilitate the integration of viral genomes into the host genome within HPV-infected cells.^41^ Recent research has found that tobacco could promote HPV-16 oncogene expression through EGFR/PI3K/AKT/C-JUN signalling pathway.^24^ Subsequently, the HPV oncoproteins E6 and E7 impede the function of p53, leading to the accumulation of chromosomal instability and the loss of cell cycle control.^41^ Ultimately, HPV-induced immortalisation, coupled with DNA damage associated with tobacco smoke, contributes to the process of carcinogenesis. In this study, we involved the covariate of smoking and confirmed a positive association between oral HPV infection and smoking.

Not only bacteria but also oncoviruses can colonise periodontitis lesions, including cytomegalovirus.^34^ As a potential risk factor, the periodontal pocket may act as a reservoir that facilitates HPV access to basal epithelial cells by disrupting the epithelial barrier. Moreover, local periodontal inflammation can further promote HPV entry into basal cells.^31^

Traditionally, periodontitis has been attributed primarily to pathogenic bacterial consortia (eg, the ‘red complex’ and other keystone organisms). However, accumulating evidence indicates that viruses – including herpesviruses and certain RNA viruses – also participate in the initiation, exacerbation, and progression of periodontitis. Increasingly, studies point to a multifaceted virus–bacterium interplay in periodontal pathogenesis.^36^ Specifically, viruses may contribute via three non-exclusive mechanisms: direct cytopathic effects, synergistic interactions with the local bacteriome, and modulation of host immune responses.^36^ For example, herpesviruses can reduce macrophage and granulocyte responses to bacterial challenges and, in addition, viral proteins expressed on infected host cells may serve as novel attachment sites for bacterial pathogens.^7,33^ Conversely, periodontal bacteria can promote viral replication and persistence through complementary routes: bacterial metabolites such as butyric acid may induce transcription of silenced genes, including the HIV1 provirus, thereby reactivating latent infection^8^; biofilm-mediated immune evasion, exemplified by virulence factors from *Porphyromonas gingivalis* (*P. gingivalis*), can dysregulate dendritic-cell apoptosis (via minor fimbriae)^29^ and is associated with reduced antiviral interferonγ responses. Together, this bidirectional synergy fosters a self-perpetuating cycle of microbial dysbiosis and epithelial barrier disruption.

The intricate relationship between vaginal microbiota and HPV has been thoroughly investigated, highlighting its crucial role in the development of cervical cancer.^30^ Therefore, exploring the interplay between oral HPV and dysbiosis of oral flora in periodontitis can help to reveal the development of oral cancer. Currently, the connection between the oral microbiome and oral HPV infection remains not fully clarified. This study indicates an increase in both species richness and phylogenetic diversity in the oral bacterial microbiota of individuals with oral HPV infection, suggesting that HPV infection could potentially influence the composition and structure of the oral microbiome. Similarly, higher richness rather than diversity was observed in HPV-positive oral swab samples compared to HPV-negative oral samples from 39 Finnish women. Additionally, unclassified *Bifidobacteriaceae* and *Finegoldia genera* were found to be significantly more abundant in HPV-positive oral samples.^37^ Another study detected a higher abundance of *Bacteroidetes phyla-Capnocytophaga ochracea* in HPV-positive chronic periodontitis granulation tissues compared to HPV-negative samples, which might be related to mismatch repair or nucleotide excision repair and cytoskeleton proteins.^9^ Furthermore, recent small-sample research found that *Prevotella intermedia*-positive cases had a higher HPV16 positivity rate.^32^ Microbiome analysis revealed distinctive characteristics in cases with deep periodontal pockets and HPV-16 infection, including higher *Porphyromonas* and lower *Veillonella* percentages, prompting further research on the detailed association with the oral microbiome.^32^ Although this cross-sectional study did not identify a significant mediation effect of the oral bacterial microbiota, this does not entirely rule out the possibility of mediation effects that may be identified in future research. Further investigation is required to clarify whether and how the oral bacterial microbiota community influences this interaction.

Indeed, the cross-sectional study design employed in this research presents inherent challenges in establishing cause-and-effect relationships or determining the directionality of observed associations. Future longitudinal studies should explore the connection between periodontitis and oral HPV infection more thoroughly. Additionally, the sample size of HPV-positive females is small (78 individuals, 3% of the female sample), which affects the statistical power and reliability of subgroup analyses. A larger sample size is necessary to draw more robust conclusions. Oral rinse samples may have influenced the positive rate of HPV DNA due to the contamination of the pharynx through gargling.^31^ Gingival tissue biopsies and crevicular fluid samples may offer a more suitable approach to ascertain the presence of HPV related to periodontal disease.^31^ Furthermore, while we utilised PCR-based HPV DNA detection – the current gold standard for mucosal infection ascertainment, the absence of serological data in NHANES failed to differentiate between transient DNA detection and immunologically confirmed infections. Future studies incorporating both oral lavage PCR and serum neutralisation assays will better characterise these relationships. In addition, sexual behaviour was not involved in this study due to the inaccessible data in NHANES. Age of first sexual intercourse, sexual activity, number of sexual partners, and types of sexual intercourse might affect the presence of oral HPV infection. Lastly, since this research focused on subjects from the NHANES 2009–2012 data set, it utilised the 2012 classification of the severity of periodontitis rather than the 2017 classification. The 2012 classification lacks the detailed staging and grading system introduced in 2017, which offers a more nuanced understanding of disease progression and associated risk factors.

Our nationally representative analysis found no overall association between periodontitis and prevalent oral HPV infection after adjustment, but identified higher odds among females with moderate to severe periodontitis and among participants with higher education. These subgroup findings, while biologically and behaviourally plausible, should be interpreted cautiously given the cross‑sectional design, potential residual confounding by sexual behaviours and vaccination, measurement limitations in both periodontal assessments and HPV detection, and the risk of false positives from multiple subgroup comparisons. Although oral HPV was associated with greater microbial diversity, diversity did not mediate the periodontitis–HPV relationship, suggesting that alternative microbial or host‑inflammatory pathways may be operative. Longitudinal studies with standardised periodontal definitions, genotype‑resolved HPV testing, and mechanistic biomarkers are needed to establish temporality and clarify modifiers of this relationship.

## CONCLUSION

In summary, no overall association was observed between periodontitis and oral HPV, but females with moderate to severe periodontitis showed higher odds of HPV detection. Oral HPV was linked to greater oral microbiome diversity without mediating the periodontitis–HPV relationship. These findings suggest no change to routine counselling for most patients, with added emphasis on periodontal care in higher-risk subgroups. Longitudinal and mechanistic studies are warranted.

## Appendix

**Table A2:** Appendix 2

Index	Brief description	Reference
Observed OTUs	Counts the number of unique operational taxonomic units (species richness)	Lozupone C, Faust K, Raes J, Faith JJ, Frank DN, Zaneveld J, Gordon JI, Knight R. Identifying genomic and metabolic features that can underlie early successional and opportunistic lifestyles of human gut symbionts. Genome Res 2012;22:1974-1984.
Faith’s phylogenetic diversity	Sum of all branch lengths in the phylogenetic tree across all species (phylogenetic richness)	Scherson R, Faith DP. Phylogenetic Diversity. Applications and Challenges in Biodiversity Science. Cham (Switzerland): Springer; 2018.
Shannon–Wiener Index	Considers both richness and evenness of species; sensitive to community complexity	Strong WL. Biased richness and evenness relationships within Shannon–Wiener index values. Ecological Indicators 2016;67:703–713.
Inverse Simpson Index	Sensitive to dominant taxa; measures diversity considering the probability that two individuals belong to different species	Kunakh O, Volkova A, Tutova G, Zhukov O. Diversity of diversity indices: which diversity measure is better? Biosystems Diversity 2023;31:131–146.


**Table A1:** Appendix 1

Categories of periodontitis	Definition
No periodontitis	No evidence of mild, moderate, or severe periodontitis
Mild periodontitis	> =2 interproximal sites with AL> =3 mm, and > =2 interproximal sites with PD > =4 mm (not on same tooth) or one site with PD > =5mm
Moderate periodontitis	> =2 interproximal sites with AL > =4 mm (not on same tooth), or > =2 interproximal sites with PD> =5 mm (not on same tooth)
Severe periodontitis	> =2 interproximal sites with AL > =6 mm (not on same tooth) and > =1 interproximal site with PD > =5 mm
Eke PI, Page RC, Wei L, Thornton‐Evans G, Genco RJ, Update of the case definitions for population‐based surveillance of periodontitis. J Periodontol 2012;83:1449–1454.
